# Super-resolved time–frequency measurements of coupled phonon dynamics in a 2D quantum material

**DOI:** 10.1038/s41598-022-22055-w

**Published:** 2022-11-17

**Authors:** Christian Gentry, Chen-Ting Liao, Wenjing You, Sinéad A. Ryan, Baldwin Akin Varner, Xun Shi, Meng-Xue Guan, Thomas Gray, Doyle Temple, Sheng Meng, Markus Raschke, Kai Rossnagel, Henry C. Kapteyn, Margaret M. Murnane, Emma Cating-Subramanian

**Affiliations:** 1 JILA, University of Colorado and NIST, Boulder, CO 80309 USA; 2 STROBE Science and Technology Center, Boulder, USA; 3 Department of Physics, Norfolk State University, Norfolk, VA 23504 USA; 4 Institute of Experimental and Applied Physics and KiNSIS, Kiel University, 24098 Kiel, Germany; 5 Ruprecht Haensel Laboratory, Deutsches Elektronen-Synchrotron DESY, 22607 Hamburg, Germany; 6 KMLabs Inc., 4775 Walnut Street, Suite 102, Boulder, CO 80301 USA; 7 Institute of Physics, Chinese Academy of Sciences, Beijing, 100190 China

**Keywords:** Characterization and analytical techniques, Optical spectroscopy, Electronic properties and materials

## Abstract

Methods to probe and understand the dynamic response of materials following impulsive excitation are important for many fields, from materials and energy sciences to chemical and neuroscience. To design more efficient nano, energy, and quantum devices, new methods are needed to uncover the dominant excitations and reaction pathways. In this work, we implement a newly-developed superlet transform—a super-resolution time-frequency analytical method—to analyze and extract phonon dynamics in a laser-excited two-dimensional (2D) quantum material. This quasi-2D system, 1*T*-TaSe_2_, supports both equilibrium and metastable light-induced charge density wave (CDW) phases mediated by strongly coupled phonons. We compare the effectiveness of the superlet transform to standard time-frequency techniques. We find that the superlet transform is superior in both time and frequency resolution, and use it to observe and validate novel physics. In particular, we show fluence-dependent changes in the coupled dynamics of three phonon modes that are similar in frequency, including the CDW amplitude mode, that clearly demonstrate a change in the dominant charge-phonon couplings. More interestingly, the frequencies of the three phonon modes, including the strongly-coupled CDW amplitude mode, remain time- and fluence-independent, which is unusual compared to previously investigated materials. Our study opens a new avenue for capturing the coherent evolution and couplings of strongly-coupled materials and quantum systems.

## Introduction

Time–frequency analysis is a critical signal processing tool in many disciplines of science and engineering. It can be used to uncover the nature of complex signals in areas spanning from communications to acoustics, radar, seismology, and neurology^1–5^ to name a few. For example, the recent detection of the first gravitational waves made use of sophisticated algorithms for time–frequency projection to characterize transient noise^6^. The need for such time–frequency representations arises from the transitory nature of signals in many systems, where the frequency or spectral components are changing over time. Under these circumstances, a simple time- or frequency-domain representation is often insufficient to capture the dynamic evolution of the system.

Two of the most commonly used time–frequency analysis tools include the short-time Fourier transform (STFT) and a continuous wavelet transform (CWT). The former was invented by Gabor in his seminal paper discussing the theory of communication^7^, while the latter was developed by Morlet et al. to solve acoustic problems in geophysics^8^. Linear prediction is also often used to extract multiple, short-lived frequency modes^9,10^. However, many materials, when laser excited, also exhibit a phonon frequency chirp (i.e. mode softening)—thus, the analysis method must be one which is capable of detecting frequency shifts in time. Recent exciting developments in advanced signal processing techniques now make it possible to extract more useful and interpretable information—which is particularly important in the case of strongly-coupled quantum materials, in which information about the coupled phonon and charge dynamics on multiple spatial (real and reciprocal space) and temporal scale lengths are critical. Gaining insight into the evolution of coupled systems can provide strategies to guide the design of functional properties and materials that are useful for next-generation nano- and quantum devices by utilizing their electronic, magnetic, optical, and thermal properties.

Very recently, a new super-resolution time–frequency analysis method called the superlet transform^11^ (SLT) was developed. The term “super-resolution” in this context is slightly different from the well-known optical super-resolution imaging—however, a common feature is the use of either structured frequency pulses (akin to structured illumination in imaging^12^) or by oversampling and averaging (akin to techniques such as STORM^13^). New approaches for metrology and scatterometry can also harness structured light^14^. Specifically, a superlet analysis uses a “structured” estimator in the time–frequency domain to better detect localized time–frequency signal components^11^. Thus, the super-resolution time–frequency analysis method can resolve the joint time–frequency density better than a single estimator can achieve, limited by the uncertainty principle.

**Figure 1:**
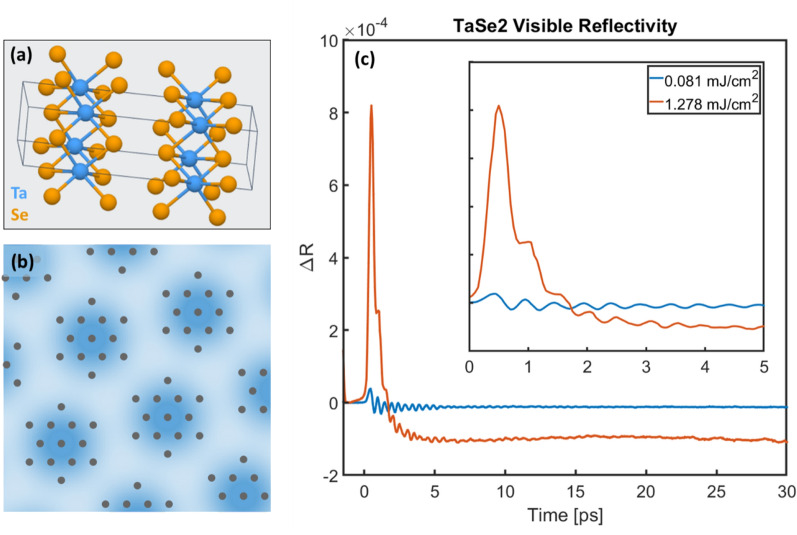
(**a**) Crystal structure of 1*T*-TaSe_2_ formed by Se-Ta-Se sandwiched layers; blue = Ta, yellow = Se. (**b**) Cartoon of the 2D CDW in 1*T*-TaSe_2_; grey dots represent the Ta atoms (atomic displacements exaggerated) while blue represents electron density (dark blue corresponding to high electron density). (**c**) Transient reflectivity of TaSe_2_ taken with high (orange) and low (blue) pump fluences. Electron excitation and relaxation are responsible for the fast rise and decay; coherent phonons are observed as an oscillating signal, and the long-lived, negative-going component comes from the change in the refractive index due to heating (incoherent phonons). Inset: the same data but only the first 5 ps are shown.

In this work, we apply this time–frequency super-resolution analysis tool to extract multiscale coupled phonon dynamics in a quasi-2D material, 1*T*-TaSe_2_, to gain a new understanding not accessible by traditional analysis tools. 1*T*-TaSe_2_ is a van der Waals material (crystal structure shown in Fig. 1a) which exhibits several phases via temperature tuning: a commensurate charge-density-wave phase (CDW, an example is shown in Fig. 1b) at temperatures below 473 K, an incommensurate CDW phase from 473 to 600 K, and a metallic ‘normal’ phase at temperatures above 600 K^15,16^. Non-equilibrium excitation, such as by a femtosecond laser pulse, has revealed the presence of additional “hidden” CDW phases with exotic properties, which lie between the usual CDW and the normal phases, and which are inaccessible through equilibrium pathways^17,18^. The strong coupling between the electrons and the lattice, which is at the root of the CDW phase, also allows for the excitation of multiple coherent phonon modes by visible light. 1*T*-TaSe_2_ is thus a promising emergent quantum material being explored in the quest for faster, lightweight, energy-efficient nanotechnologies. However, its inherent complexity makes a full understanding of electron–phonon coupling in this system very challenging.

**Figure 2:**
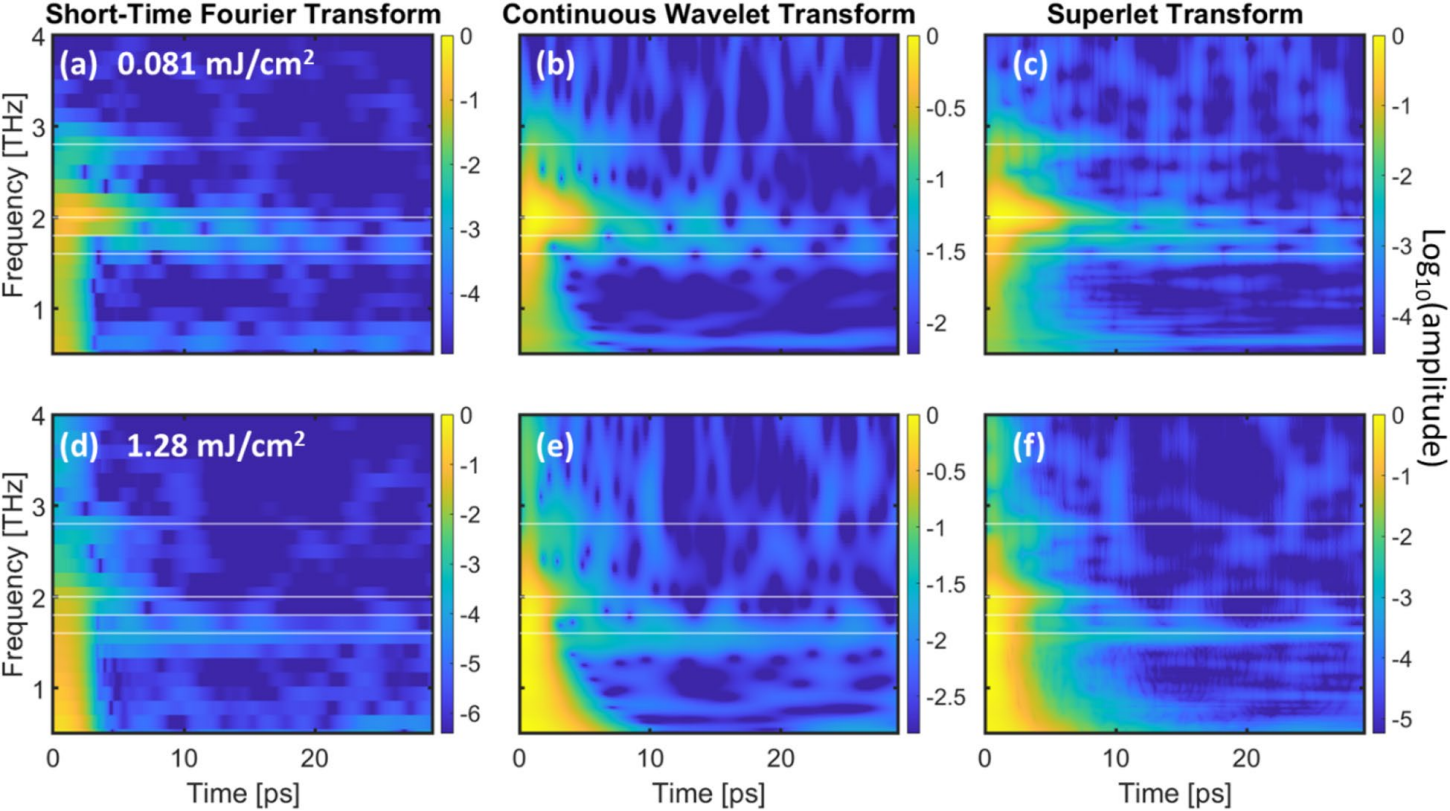
Comparison of the Short-Time Fourier Transform (STFT), Continuous Wavelet Transform (CWT), and Superlet Transform (SLT) for 0.081 mJ/cm^2^ pump fluence (**a**–**c**, respectively) and for 1.278 mJ/cm^2^ pump fluence (**d**–**f**, respectively). White lines at 1.6, 1.8, 2, and 2.8 THz are guides to the eye. All transforms use a normalized Log_10_ color scaling for ease of comparison. The SLT outperforms the other two techniques in both spectral and temporal resolution.

Experimentally, we used an ultrashort laser pulse at a wavelength of 800 nm to excite coherent phonons in this material, and then probed the resultant excitations by reflecting a delayed 400 nm probe beam off of the sample. Utilizing the SLT^11^ technique to analyze the reflectivity data, we are able to distinguish three phonon modes at 1.6, 1.8, and 2.0 THz, which would otherwise be too close in frequency and too short-lived to be resolved (see Fig. 2). With enhanced resolution in both the time and frequency domain, we are able to track the temporal evolution of these phonon frequencies. Surprisingly, unlike observations in other laser excited materials where phonons were observed to soften with both time^19^ and pump fluence^20,21^, the frequencies of all three phonon modes stay the same, indicating unusual light induced electronic structures and interatomic potential. As the pump laser fluence is changed, we see the relative intensities of these three phonon modes change, and we track this shift to gain an understanding of the fluence-dependent coupling between the electron and phonon systems. Previous trARPES (time- and angle-resolved photoemission spectroscopy) studies of 1*T*-TaSe_2_ showed what was speculated to be a mode-selective electron–phonon coupling^18^. The quasi-equilibrium electron and phonon temperature measured by trARPES at 4 ps increased with laser fluence up to 0.7 mJ/cm^2^, above which the temperature increased abruptly, associated with a decrease in effective lattice heat capacity by about 70%. This decrease in heat capacity suggests that above the critical fluence, only a small subset of the phonon modes are initially strongly coupled to the electron bath. The data presented here aligns with and validates this interpretation, as we see a distinct change in the relative intensities of the strongly-coupled modes as a function of laser fluence (Fig. 2). This allows us to directly observe a previous speculation^17^ that suggests that there could be a dominant direct electron-coherent-phonon coupling at high fluence, resulting in a strongly-damped 2 THz mode.

## Results and discussion

We studied photoexcited phonon modes in 1*T*-TaSe_2_ by monitoring the transient reflectivity of a laser pulse following impulsive excitation by a femtosecond laser (see “Methods”). Briefly, we used a 60 fs, 800 nm laser pulse to photoexcite electrons in the material. A second probe laser pulse (60 fs, 400 nm) was used to interrogate the sample at various time delays by monitoring the intensity of the reflected blue light (Fig. 1c). The transient reflectivity changes are due to the presence of the excited electrons and phonons that modify the overall refractive index of the sample. The initial fast rise and exponentially-decaying signal is due to the excitation and subsequent relaxation of hot electrons. Upon laser excitation, some electrons are excited into delocalized unoccupied states, which smears out the spatial charge modulation and coherently generates phonons through displacive excitation^17^. Within ~ 30 fs, electron–electron scattering establishes a hot Fermi distribution^17^. These hot electrons then relax via electron–phonon and phonon–phonon scattering to different phonon baths, e.g., one set of strongly-coupled modes that depend on the laser fluence (both coherent and incoherent), and very-weakly coupled modes that do not participate for tens of picoseconds to nanoseconds. The strongly-coupled coherent phonon modes show up as an oscillating signal that is particularly strong for timescales shorter than 5 ps (Fig. 1c, inset), while incoherent phonons are responsible for the long-lived negative-going baseline that is clear after about 1–3 ps.

Previous Raman and transient reflectivity experiments as well as density function theory simulations have shown that 1*T*-TaSe_2_ supports a complex phonon spectrum with many modes close to one another in frequency (Figure SI.1; Table SI.T1)^16,22^. In the transient reflectivity data, these modes appear to beat off one another and quickly damp and decohere. At longer times (~ 30 ps), the oscillations have largely damped and the long-lived negative-going signal results from incoherent phonons similar to what equilibrium heating of the material would produce.

The presence of multiple decaying modes that are close together in frequency and have short, yet varied, lifetimes limits the usefulness of simple time–frequency techniques like STFT and necessitates a more sophisticated approach. The STFT uses windowed sine functions to build up the ultimate spectrum and requires spectral components to be approximately constant over the time window used. However, the decay rate of some modes in our experiment is quite short relative to their period, making STFT ineffective. The STFT has another drawback—its time–frequency window is fixed over all frequencies and time spans, in other words, a uniform “sampling window” on a time–frequency representation. Because low frequencies tend to last longer, a higher frequency and lower time resolution is ideal in these regions, while the faster-decaying high frequencies prefer a high time and low frequency resolution.

Another commonly used time–frequency analysis tool over the past three decades is the CWT^23,24^. Instead of using a fixed time–frequency window, a wavelet transform uses a fixed number of cycles at the probed center wavelengths. Rather than using a set of windowed sine or cosine waves to construct the signal, CWT uses a more complex set of orthonormal waveforms. Because any orthonormal functions can be used, the wavelet transform is somewhat tunable, letting the user select waveforms that are most well-suited to the problem. A typical choice is known as a Morlet wavelet, but there are several common bases^8,25^. Importantly, the resolution of a wavelet transform varies with frequency, providing a high frequency and low time resolution for low frequency components, and low frequency and high time resolution for high frequency components. In other words, CWT uses uneven sampling windows (or grids) to provide a frequency-dependent time resolution. While this can improve on the STFT, the wavelet transform is still Gabor limited—not ideal in cases such as 1*T*-TaSe_2_ where there are several frequency components that are quite close together in time and decay quickly.

The SLT, a super-resolution technique recently developed by Moca et al.^11^, combines sets of wavelet transforms with varying time–frequency resolutions. It combines short wavelets which have excellent time but poor frequency resolution with progressively longer wavelets (more cycles) that have increasingly better frequency resolution. In a time–frequency representation, a superlet sampling window or grid presents a weighted, cross-like shape on the 2D time–frequency plane, analogous to the types of “structured illumination” that enable super-resolution in 2D imaging and microscopy. The principle of the SLT is similar to the existing minimum mean cross-entropy techniques^26,27^, which combine multiple STFTs to produce a super-resolution time–frequency representation. However, the selection of parameters for the superlet is much simpler than for minimum mean cross-entropy techniques, which require a careful selection of window sizes. Compared to traditional methods (STFT and CWT), the SLT excels at detecting frequency components near strong neighbors in both frequency and time.

To demonstrate the power of this new technique, we generated a set of time–frequency spectrograms using the SLT, STFT, and a Morlet CWT from experimentally measured transient reflectivity data. Figure 2 shows the STFT (left), CWT (middle), and SLT (right) spectrograms for data at both low (top) and high (bottom) pump laser fluences. The spectral intensity is plotted on a log scale to highlight the long-lived components. There are several frequency components present, with the most dominant and obvious being those at 2.8, 2, 1.8, and 1.6 THz. Note that the spectral resolutions of the STFT and the CWT are too poor to clearly distinguish the 1.6, 1.8, and 2 THz modes from each other.

Comparing the low and high laser fluence SLT spectrograms, we can see that the 1.6, 1.8, and 2 THz modes have different relative intensities and lifetimes in the high and low fluence regimes. This may indicate a fluence-dependent shift in the electron–phonon or phonon–phonon couplings, as speculated by Zhang et al.^17^, which cannot be resolved by other time–frequency techniques. Previous trARPES experiments on this material showed that the electronic and the structural orders are strongly coupled^17^. They also observed a phase change of π in the electron temperature modulation at a critical fluence of 0.7 mJ/cm^2^, which suggests a switching of the dominant coupling mechanism between the coherent phonon and electrons. Indeed, Zhang et al*.* speculated about the presence of a dominant *direct* electron-coherent-phonon coupling at high fluence, and a dominant coupling involving more phonons at low fluence.

We see similar behavior in the transient reflectivity. Our low-fluence regime is substantially below the critical fluence (0.7 mJ/cm^2^) while our high-fluence regime is above the critical fluence as we show in Figure SI.2. In the low-fluence SLT, the 2 THz and 1.8 THz modes look stronger and persist longer than the 1.6 THz mode. At high fluences, the 1.6 THz and 1.8 THz appear stronger than they did at low fluence, and persist significantly longer, while the 2 THz mode decays more quickly than it did at low fluence. This indicates that the couplings which both populate (leading to mode intensity) and depopulate (leading to mode decay) the three modes have changed in their relative strength as a result of changing the fluence. It is also worth noting that the effect may be quite a bit stronger than it appears to be from our data. Our sample is many tens of microns thick, and while the optical parameters of 1*T*-TaSe2 are not fully characterized, we estimate the absorption depth to be approximately 25–30 nm for both wavelengths based on calculations^28^ by Reshak et al. This means that the change in probe reflectivity is due to an average of the layers it interacts with, and at high pump fluence the probe actually interacts with some layers which have seen high fluence, and some which have seen lower fluences. Therefore, we would expect the difference between the surface-only reflectivity to be larger than what we observe here.

While the SLT is a powerful technique, some caution must be taken when interpreting the results. Figure 2 shows what seems to be an out-of-phase intensity modulation between the 3 modes centered at 1.8 THz at low fluence. However, this is simply an artifact of the transform. Beat patterns in the original signal show up as a periodic modulation of the mode intensity in these types of transforms (see Supplemental Information Fig. SI.3).

### Analysis of the technique

**Figure 3:**
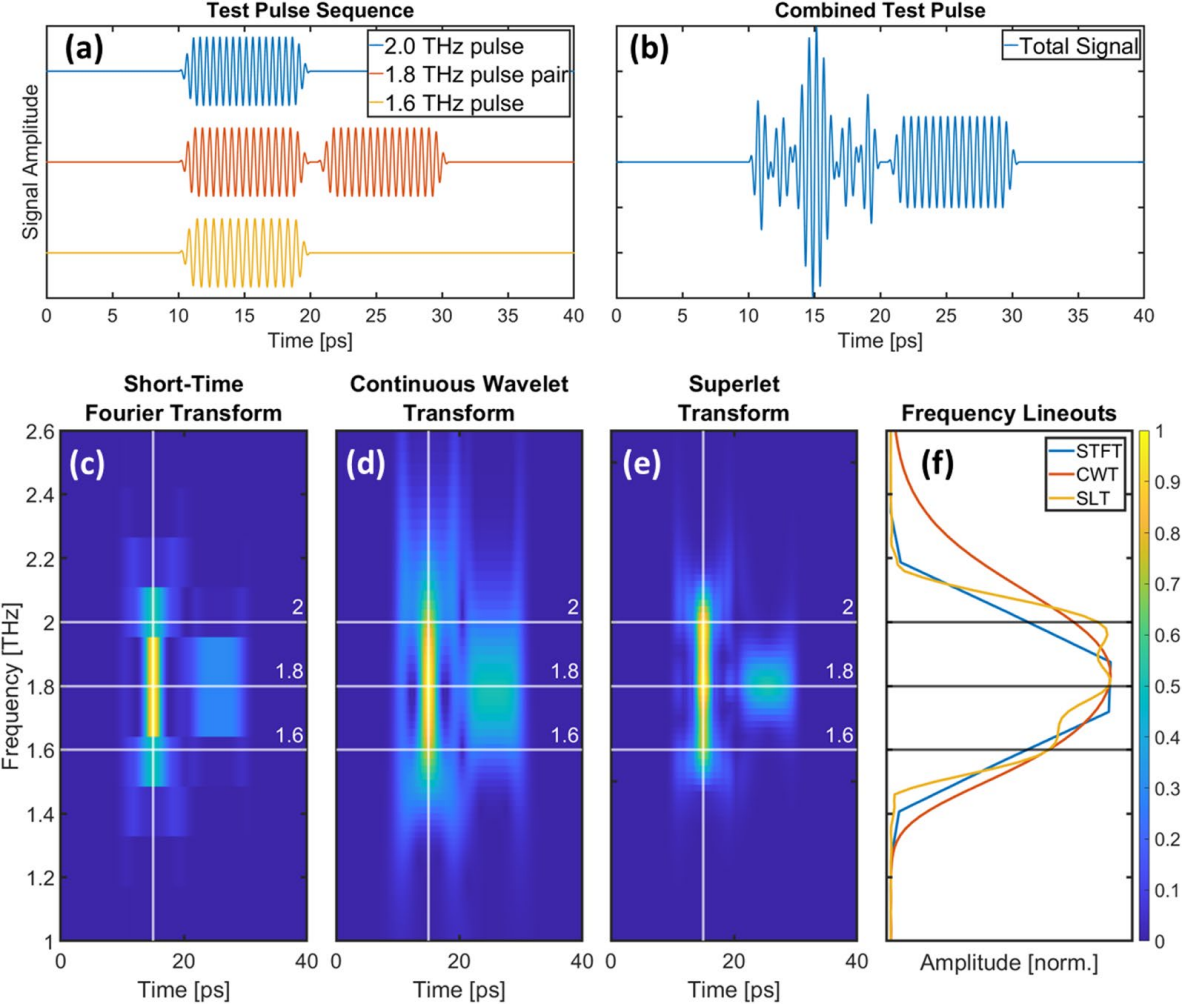
Demonstration of SLT performance using test pulse series. (**a**) Test pulses with frequency components 2 THz, 1.8 THz, and 1.6 THz, are summed to generate the model signal in panel (**b**). Each pulse is 10 ps in duration and the 1.8 THz component has an additional pulse delayed in time by 0.5 ps from the end of the first pulse. This model signal will let us study the temporal and spectral resolution of the three techniques. The normalized STFT (**c**), CWT (**d**), and SLT (**e**) of the model signal are shown in the bottom panels from left to right. Lineouts (**f**) of each transform taken at 15 ps (denoted by the vertical white lines in the transforms) are shown in the bottom right panel. The additional structure present in the spectral lineout of the SLT demonstrates its improved spectral resolution. Colorbar indicates relative intensity (arb. units).

**Figure 4:**
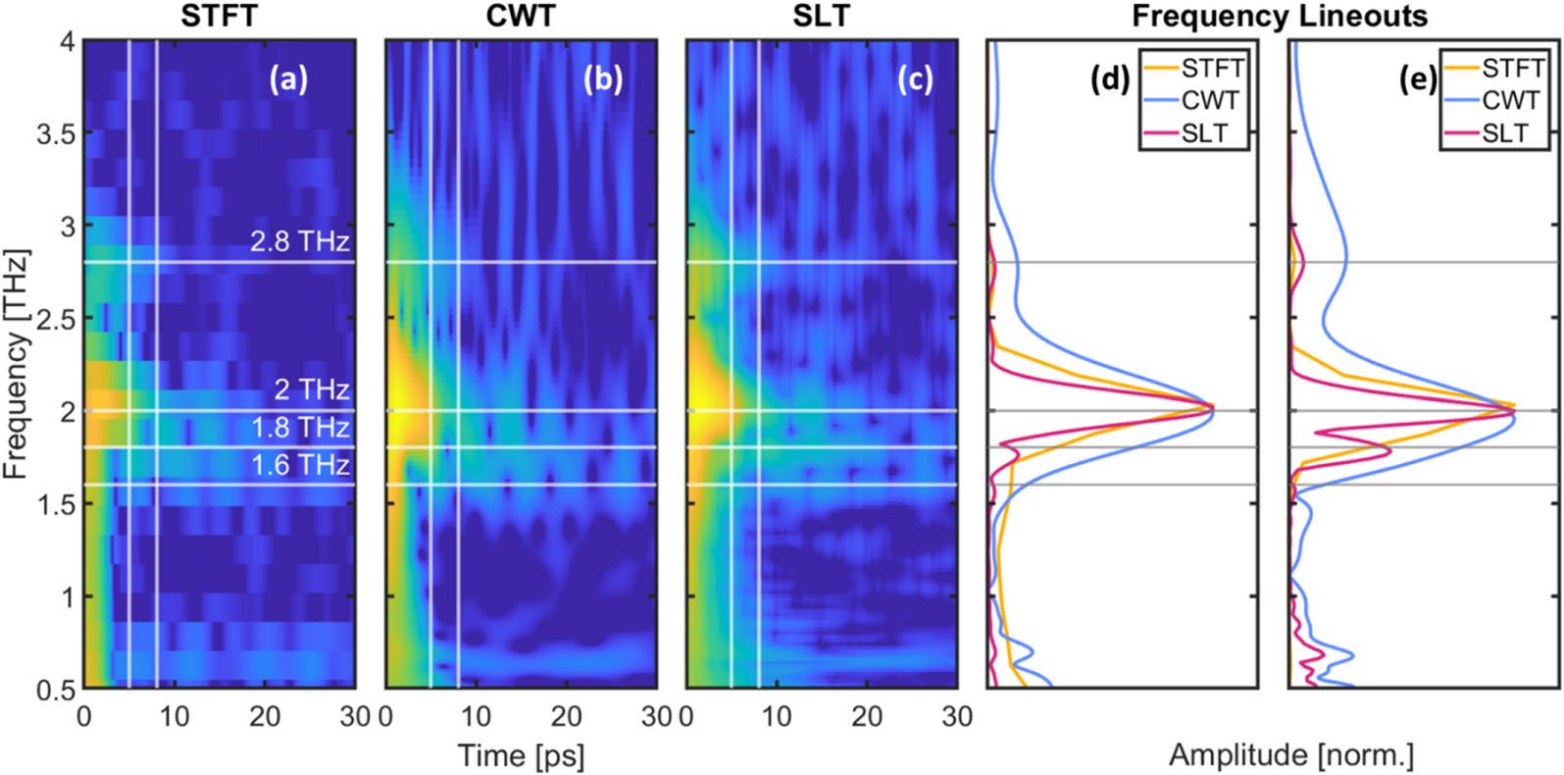
Demonstration of SLT performance using real data taken with 0.081 mJ/cm^2^ pump fluence. (**a**–**c**) STFT, CWT, and SLT spectrograms, respectively. Lineouts were taken at 5 ps (**d**) and 8 ps (**e**) indicated by the vertical white lines in the spectrograms. Horizontal lines at 1.6, 1.8, 2, and 2.8 THz serve as a guide to the eye. The lineouts show that the SLT produces a narrower peak than either alternative, and provide better spectral resolution. The peak at 1.8 THz is not resolved by the CWT or STFT (blue, yellow, respectively), but is clearly present in the SLT lineout (red) at both 5 and 8 ps.

To demonstrate the enhanced ability of the SLT to distinguish different signals, we modeled three pulsed phonon signals (Fig. 3a: model of ground truth signals, which are usually unknown to the experimenters), which interfere to produce a complicated transient reflectivity signal (Fig. 3b: signal A + B + C, which is what an experiment can measure). To make sense of this summed waveform, we need a way of extracting or retrieving the original signals A, B, and C. This is made more challenging by the fact that the amplitudes all vary in time. Therefore, a time–frequency analytical technique is needed to retrieve the original signals. Figure 3c–e show a set of time–frequency representations, so-called spectrograms, derived from performing an STFT, CWT, and SLT on the resulting waveform. The parameters of the STFT and CWT were chosen such that they maintained a similar time resolution as the SLT. As can be seen by the vertical smearing in the STFT and CWT, and the corresponding frequency lineouts in Fig. 3f, these techniques have worse frequency resolution as compared to the SLT. The same effect can be seen in the real data. Figure 4 shows three spectrograms (STFT, CWT, and SLT) of the 0.081 mJ/cm^2^ pump fluence data as well as frequency lineouts from 5 and 8 ps. At both times the SLT produces narrower frequency peaks than either of the other two methods, and is the only method that can distinguish the 1.8 and 2 THz peaks.

To quantify the resolution of the superlet technique and compare it to other time–frequency methods, we numerically conducted an impulse response analysis. We generated a set of delta functions in time and then in frequency as our impulses, then passed them through each of our transforms which serve as our dynamic systems. The transforms necessarily spread the inputs due to their inherent uncertainty, resulting in impulsive response functions analogous to point spread function analysis in 2D imaging. This spread is measured by fitting it with a Gaussian and extracting the standard deviation. The results of the time–frequency uncertainty comparison are summarized in the Supplementary Information, Fig. SI.4. The SLT consistently outperforms the CWT over our entire frequency window (0.5–4 THz): the temporal uncertainty in the SLT is approximately 1.75 times better than the temporal uncertainty of the CWT, while the frequency uncertainty of the SLT is approximately 3.3 times better than the CWT over the same range. The uncertainties in frequency and time are related to frequency linearly and inversely, respectively, for both the CWT and SLT. The STFT on the other hand has both temporal and frequency uncertainties which are constant over frequency. This means that there are regions where it can outperform the SLT in either frequency resolution (high frequencies) or temporal resolution (low frequencies), but never both simultaneously.

## Conclusion

Time–frequency analysis techniques are common throughout many areas of science and engineering. Often, in order to resolve frequency components that are close together and exhibit short lifetimes, one must increase the coherence lifetimes in a material by lowering the temperature^22^, or deliberately damp specific modes using a second pump pulse as in pump-pump-probe experiments^29^. The improved time–frequency resolution of the SLT opens up possibilities for performing experiments on oscillatory signals at room temperature and with simpler experimental design. Because it is generally applicable to multi-component oscillating signals, the SLT will undoubtedly find use in a wide variety of experimental settings and techniques and help provide a deeper understanding of dynamics and couplings in quantum materials.

Using 1*T*-TaSe_2_ as a model system, we demonstrate what is to our knowledge the first application of the SLT to a physical system, and its benefits over standard time–frequency analysis methods. The SLT allowed us to distinguish three quickly-decohering modes that the STFT and CWT could not resolve. We could then monitor the pump-fluence-dependent relative intensity of the three modes and gained an improved understanding of the electron-phonon coupling in the CDW, normal, and hidden phases of 1*T*-TaSe_2_. We observed that, contrary to expectations, the phonon frequencies do not change with time or laser pump fluence. We also confirmed a hypothesis proposed by Zhang et al*.*^17^ that pump fluence induces changes in the dominant electron–phonon couplings. Due to the general use of time–frequency measurements to investigate dynamic systems, we also anticipate that our study will stimulate the community to reexamine their existing time series data—which could unveil overlooked signals in condensed matter physics, materials science, and beyond.

## Methods

### Transient reflectivity

An ultrafast laser “pump” pulse (800 nm, 60 fs, 100 kHz) was focused to a 35 µm diameter spot on the 1*T*-TaSe_2_ sample. A second pulse, the probe, was frequency doubled to 400 nm and focused to a 26 µm diameter spot on the sample, interrogated its excited state, and the reflected probe was detected by a balanced photodiode and lock-in amplifier. Both pump and probe beams were at near-normal incidence to the TaSe_2_ surface. A delay stage in the pump path was used to control the relative arrival time of the pump and probe pulse, and an optical chopper in the pump path blocked half of the pump pulses, allowing the probe to interrogate both the excited and ground states of the material. Finally, a lock-in amplifier determined the difference in reflected light between pump-on and pump-off states. We used a pump fluence of 0.081 mJ/cm^2^ for the low fluence regime, and 1.28 mJ/cm^2^ for the high fluence regime. The dynamics were consistent regardless of beam polarization.

### Superlet transform implementation

One of the additional benefits of the superlet transform (SLT) is the approachability and simple choice of parameters for optimizing the resulting time–frequency representation. Fourier-based minimum mean cross-entropy techniques^26,27^ are a set of established time–frequency super-resolution methods, but they suffer from a window-size selection problem that must be manually tested. When parameter choices are sub-optimal, there will be excessive time/frequency smearing. Directionally smoothed Wigner–Ville distributions offer the highest possible time–frequency resolution^30^, but suffer from cross-terms in multi-component signals^31^. A variety of smoothing kernels can be applied to the Wigner–Ville distribution leading to an infinite number of time–frequency representations, and once again, the selection of kernel(s) is not always intuitive.

On the other hand, optimization of the SLT is intuitive and approachable. There are 3 parameter choices involved with implementing the superlet: base cycle number *N*_*c*_, order *O*, and the scaling of the set of wavelets (either additive or multiplicative). The base cycle number sets the maximum time-resolution; by reducing *N*_*c*_ the time-resolution is increased. The order of the superlet determines the number of wavelets used to create the resultant spectrogram. The wavelets scale in size either additively {*N*_*c*_, *N*_*c*_ + 1, *N*_*c*_ + 2…, *N*_*c*_ + O} or multiplicatively {*N*_*c*_, 2*N_*c*_, …, O*N_*c*_}. Increasing the order of the superlet increases the frequency resolution of the time–frequency representation. Multiplicative scaling is most useful when attempting to span both high temporal and high frequency resolution where an additive scaling would require the order parameter to be excessively large. The base cycle number used in this study is ${N}_{c}=3$, the order of the superlet is 11, and we used a multiplicative scaling for cycle numbers. This means that our superlet used a set of 11 wavelets with cycle numbers $\left\{\mathrm{3,6},9,\dots ,33\right\}$. The wavelet transforms were then combined using a geometric mean to produce the SLT.

### Other time–frequency representations

We compared two standard transformation methods to the SLT: a Short-Time Fourier Transform (STFT) and a Continuous Wavelet Transform (CWT) using Matlab’s built-in functions (stft() and cwt(), respectively) from the Signal Processing Toolbox and Wavelet Toolbox. The parameters for these control transforms were chosen such that the temporal and frequency uncertainty near 1.8 THz are approximately equal (see SI Fig. 4). The STFT in this paper uses a window length of 6.4 ps and a periodic Hanning window function. The CWT uses a Morse wavelet (Morlet)^8^ with a time-bandwidth product of 120 and a symmetry parameter of 3, corresponding to a symmetric, unskewed wavelet.

### First-principles vibrational mode simulations

The first-principles calculations were performed on the basis of density functional theory (DFT) as implemented in the Vienna ab initio simulation package (VASP) software^32^. Bulk 1*T*-TaSe_2_ in its CDW state was simulated with a supercell of 78 atoms with √13 × √13 × 2 periodical boundary conditions. The generalized gradient approximation (GGA) proposed by Perdew, Burke, and Ernzerhof (PBE) is used as the exchange–correlation functional^33^. Ion cores were described by using the projector augmented wave (PAW) method^34^. A 3 × 3 × 1 k-point grid generated by Monkhorst–Pack was used in geometry relaxation, and the relaxation would only stop when the maximal Hellmann–Feynman force of all atoms was less than 10^−3^ eV/Å and free energy was less than 10^−5^ eV. The energy cutoff for the expansion of the plane wave basis was set to be 400 eV. To calculate the phonon vibrational frequencies and eigenvectors at zone center, finite differences were used.

## Supplementary Information

Supplementary Information.

## Data Availability

The datasets utilized to prepare the data presented in this manuscript are available and free of charge from the corresponding author under reasonable request.
