# Author Correction: Super-resolved time–frequency measurements of coupled phonon dynamics in a 2D quantum material

**DOI:** 10.1038/s41598-023-37989-y

**Published:** 2023-07-04

**Authors:** Christian Gentry, Chen-Ting Liao, Wenjing You, Sinéad A. Ryan, Baldwin Akin Varner, Xun Shi, Meng-Xue Guan, Thomas Gray, Doyle Temple, Sheng Meng, Markus Raschke, Kai Rossnagel, Henry C. Kapteyn, Margaret M. Murnane, Emma Cating-Subramanian

**Affiliations:** 1 JILA, University of Colorado and NIST, Boulder, CO 80309 USA; 2 STROBE Science and Technology Center, Boulder, USA; 3 Department of Physics, Norfolk State University, Norfolk, VA 23504 USA; 4 Institute of Experimental and Applied Physics and KiNSIS, Kiel University, 24098 Kiel, Germany; 5 Ruprecht Haensel Laboratory, Deutsches Elektronen-Synchrotron DESY, 22607 Hamburg, Germany; 6 KMLabs Inc., 4775 Walnut Street, Suite 102, Boulder, CO 80301 USA; 7 Institute of Physics, Chinese Academy of Sciences, Beijing, 100190 China

Correction to: *Scientific Reports* 10.1038/s41598-022-22055-w, published online 17 November 2022

The Acknowledgments section in the original version of this Article was incomplete.

“The US authors gratefully acknowledge support from a DOE Basic Energy Sciences Grant DE-FG02-99ER14982 for the experimental setups and measurements, the Physics Frontier Center grant PHY1734006 for the correlative ARPES measurements, and STROBE STC Grant (NSF DMR 1548924 for supporting the collaboration between NSU and CU Boulder. We thank Dr. Michael Gerrity for his laser expertise and aid and Yingchao Zhang for his thoughtful insights.”

now reads:

“The US authors gratefully acknowledge support from a DOE Basic Energy Sciences Grant DE-FG02-99ER14982 for the experimental setups and measurements, the Physics Frontier Center grant PHY1734006 for the correlative ARPES measurements, STROBE NSF STC grant and PEAQS NSF PREM grant (NSF DMR 1548924 and NSF DMR 1827847 for supporting the collaboration between NSU and CU Boulder). We thank Dr. Michael Gerrity for his laser expertise and aid and Yingchao Zhang for his thoughtful insights.”

The original Article has been corrected.

