# Supplementary material for: Super-resolved time–frequency measurements of coupled phonon dynamics in a 2D quantum material
Source: Sci Rep. 2022 Nov 17;12:19734. doi: 10.1038/s41598-022-22055-w (PMC9672042; doi:10.1038/s41598-022-22055-w)
Supplement: Supplementary file 1 — Supplementary Information. [file 41598_2022_22055_MOESM1_ESM.pdf]

Supplementary Information for "Super-resolved time-frequency measurements of coupled phonon dynamics in a 2D quantum material"

A phonon spectrum (Figure SI.1, top left), vibrational vectors (Figure SI.1 right), and frequencies (Table SI.T1) for 1*T*-$TaSe_2$ were calculated using DFT (density functional theory) and a large supercell of 78 Ta atoms. Table SI.T1 contains a representative set of the more than 200 vibrational frequencies identified. Many of the frequencies were within less than 0.01 THz of each other. We cannot resolve such small increments in our data, therefore, each mode we observe may be multiple modes very close in frequency.

For bilayer 1*T*-$TaSe_2$, we found that the phonon mode at the Γ point (frequency ~2.49 THz) is similar to the CDW breathing mode (2.0 THz in the experiment). The small mismatch in frequency might be attributed to the choice of the simulation method and accuracy. For a large supercell with 78 atoms, it is hard to adopt a higher-precision strategy due to the excessive computational cost. Nonetheless, there are only three imaginary frequencies with their values near zero, implying that the simulation is reasonable for a loose standard.

Figure SI.1 shows the vibration vectors (arrows) of the Ta atoms (blue dots). There is a ~0.5 THz difference in the measured and calculated CDW breathing mode frequency. We identified our 2 THz mode as the breathing mode by looking at the band shift in ARPES data (References 15, 16). When choosing the phonon mode to show in figure SI.1 we assumed that the frequency shift between experimental and calculated data would be similar for the other modes as it is for the breathing mode (~ 0.5 THz).

We see critical behavior at a pump fluence above about 0.7 mJ/cm^2: at lower fluences, the ΔI value at 10 ps decreases with increasing fluence, but higher fluences show approximately the same ΔI value at 10 ps (Figure SI.2, left). In Figure SI.2., right, we show the average signal from 3.5 – 4 ps as a function of fluence. We averaged the signal over 500 fs to mitigate the effect of the oscillations in the signal, and chose to look at the signal at 3.5 – 4 ps so we could directly compare this with the trend observed in Reference 16, in which the electron temperature plateaued at 4 ps. There is a sharp kink in the ΔI right around 0.7 mJ/cm^2, indicating critical behavior.

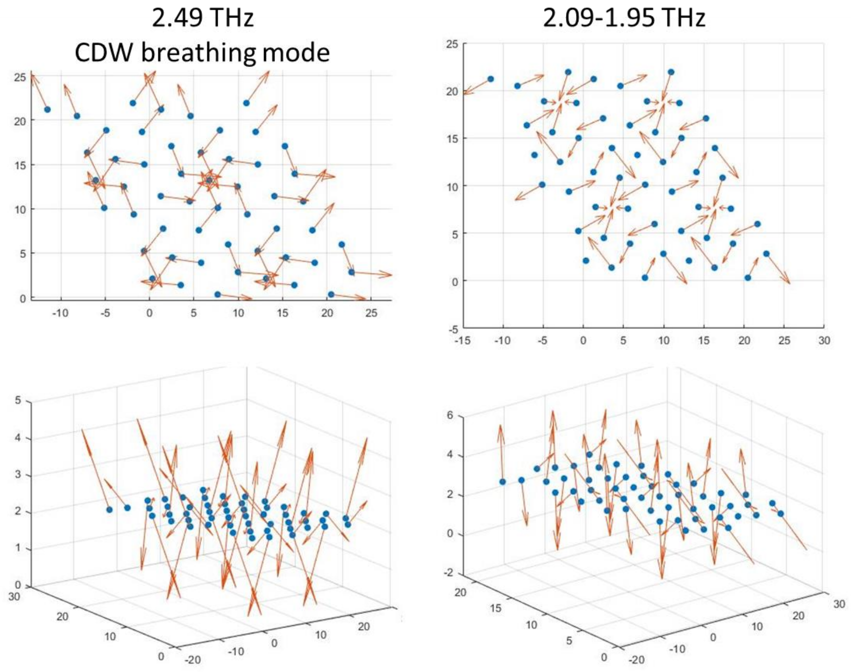

Figure SI.1. Locations of atoms (blue dots) and momentum vectors (orange arrows) for the CDW breathing mode (center, 2.5 THz corresponding with measured 2.0 THz mode) and an average of modes from 2.09 THz to 1.95 THz (right, corresponding with measured 1.6 THz mode).

**THz**	**cm^{-1}**
2.97	99.0
2.81	93.7
2.79	93.0
2.71	90.5
2.67	89.1
2.09	69.8
1.98	65.9
1.95	63.7
1.82	60.7
1.56	52.2
1.48	49.4

Table SI.T1: Representative set of calculated phonon modes for 1*T*-$TaSe_2$.

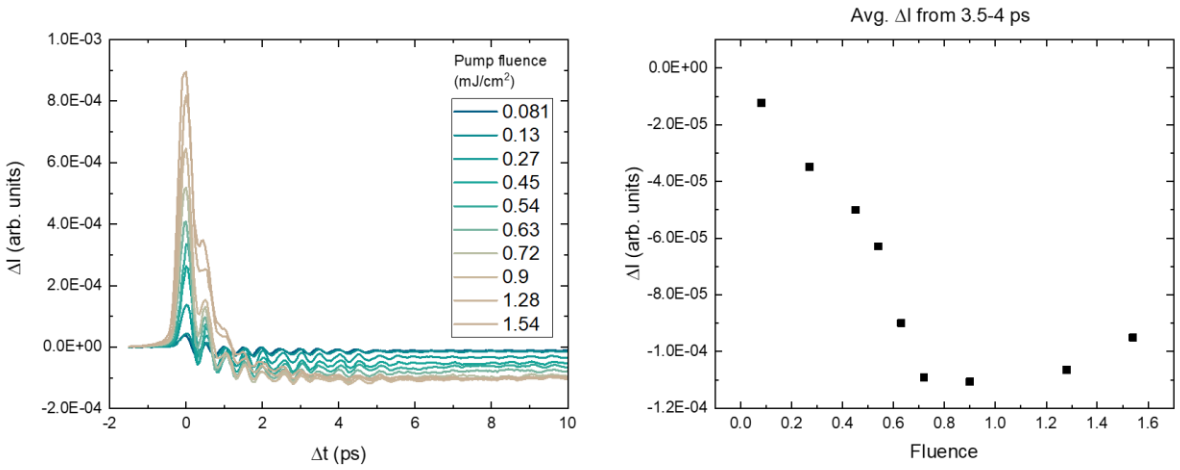

Figure SI.2. Left: Transient reflectivity scans from -1 to 10 ps taken with fluences from 0.081 mJ/cm^2 to 1.54 mJ/cm^2. Note that the ΔI at later times tends to become more negative with increasing fluence up to around 0.7 mJ/cm^2. Right: Average ΔI between 3.5-4 ps versus fluence. Note the inflection point of the trend at 0.7 mJ/cm^2 indicating critical behavior.

Continuous wavelet transforms (CWTs) and the superlet transform (SLT) act as instantaneous power filters. When two or more signals (waveforms) are present and coherent with each other, they will interfere. When these patterns destructively interfere, there is no instantaneous power present in the signal. The SLT will represent this as a loss of intensity in those frequency components. These artifacts in the time-frequency representation can be demonstrated with two constant amplitude waves. As frequency components are added to the signal, additional beat patterns and cross-terms may form, leading to a significantly complicated structure in the SLT. One benefit of these artifacts is that they can reveal the relative phase between coherent signals. By phase shifting a frequency component relative to the others, the structure within the superlet transform changes, specifically the apparent minima shift in time, as seen in Figure S1.3.

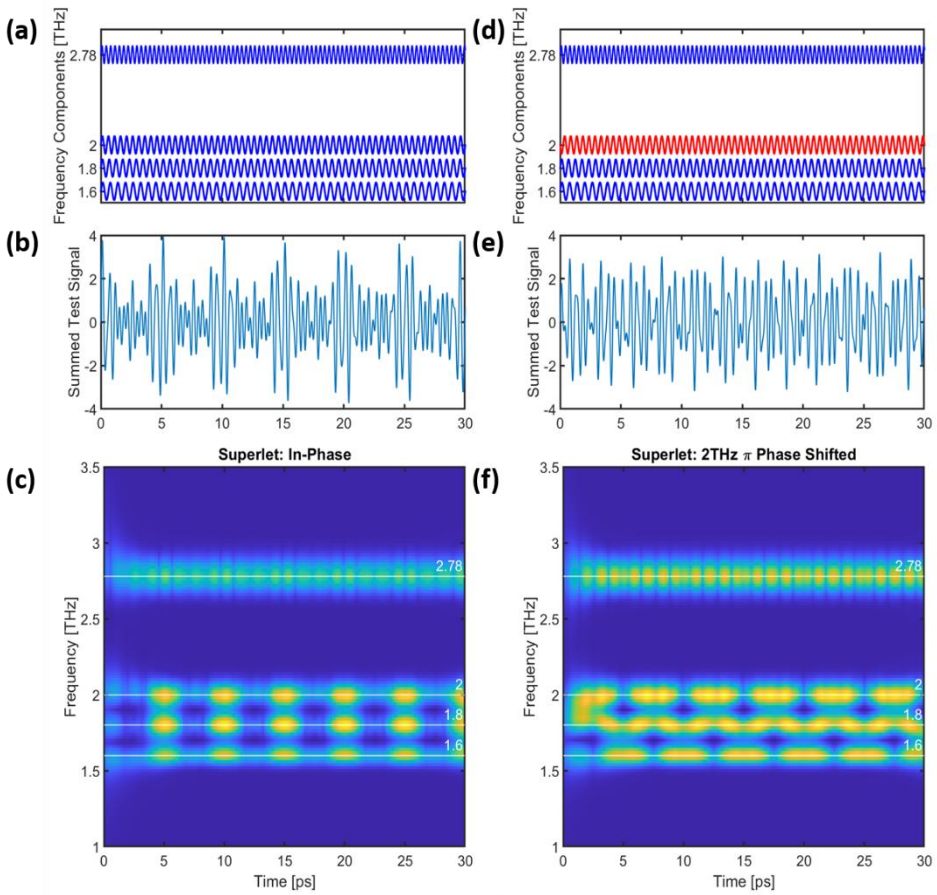

Figure SI.3. Beat frequency and transform artifacts. (a) Time domain of each frequency component: 2.78 THz, 2 THz, 1.8 THz, and 1.6 THz. Each frequency is in phase with each other. (b) Sum of frequency components in (a). (c) The SLT of in-phase frequency components demonstrates the effect of beat patterns on the resulting transform. (d) Time domain of each frequency component with 2 THz component π phase shifted. (e) Sum of frequency components in (d). (f) SLT of frequency components with 2THz component π phase shifted relatively, demonstrating the effect of beat patterns and cross-terms.

It is critical to characterize the uncertainty in both time and frequency when performing time-frequency analysis. With transient, multi-component signals, this is notoriously difficult, and we leave the details to the authors of the original paper (Reference 9). Here, we characterize the relative uncertainty between the STFT, CWT, and SLT. Temporal uncertainty is calculated by passing a delta function in time $\delta(t)$ through each transform; the transforms inherently spread that signal which is then fitted with a Gaussian. The standard deviation (STD) of the Gaussian is the metric used for time uncertainty. A similar process is

used to determine the frequency uncertainty, except a delta function at each probed frequency $\delta(f - f_i)$ is passed into each transform. Fits of the temporal and frequency uncertainty curves yield:

$$\Delta T_{CWT} = 1.75/\nu \quad (1) \qquad \Delta F_{CWT} = 0.0916 * \nu + 1.6 * 10^{-4} \quad (2)$$

$$\Delta T_{SLT} = 1/\nu \quad (3) \qquad \Delta F_{SLT} = 0.0277 * \nu + 2.1 * 10^{-4} \quad (4)$$

$$\Delta T_{STFT} = 0.96 \quad (5) \qquad \Delta F_{STFT} = 0.09\ (\text{approx.}) \quad (6)$$

where ν is a known input frequency in THz, ΔT is the temporal uncertainty in ps, and ΔF is the frequency uncertainty in THz. The results of these equations are plotted in Figure SI.4. The wandering of the frequency uncertainty in the STFT is due to sparseness of frequency sampling. The time and frequency uncertainty of the STFT and CWT matches extremely well with the theoretical values expected from the standard deviations of the window function and wavelet, respectively.

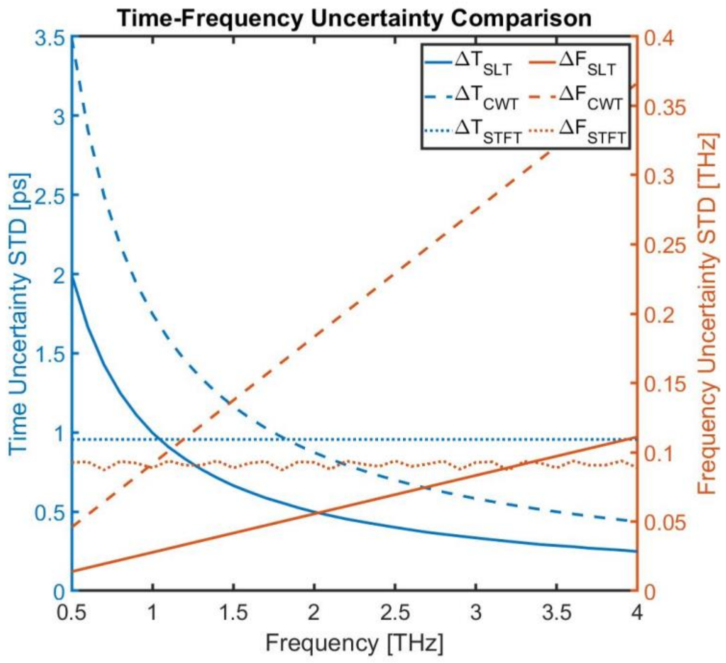

Figure SI.4. Time-frequency uncertainty comparison. Time and frequency uncertainties for the SLT, CWT, and STFT.
